# Tribological Properties of Carbon Fabric/Epoxy Composites Filled with FGr@MoS_2_ Hybrids under Dry Sliding Conditions

**DOI:** 10.3390/ma15227951

**Published:** 2022-11-10

**Authors:** Wen Zhong, Siqiang Chen, Lei Ma, Zhe Tong

**Affiliations:** 1 The Key Laboratory of Fluid and Power Machinery, Ministry of Education, Xihua University, Chengdu 610039, China; 2 Luzhou Laojiao Group Co., Ltd., Luzhou 646000, China; 3 School of Mechanical Engineering, North University of China, Taiyuan 030051, China

**Keywords:** CFRP, FGr@MoS_2_ hybrid, dry sliding, self-lubricating, temperature

## Abstract

Hybrids of fluorinated graphite/MoS_2_ (FGr@MoS_2_) were prepared via a hydrothermal method and used as lubricating additives to take full advantage of the synergy between FGr and MoS_2_ in carbon-fiber-reinforced polymer (CFRP). The results show a 21.6% reduction in the friction coefficient compared to the neat sample when the CFRP was filled with 1.2 wt.% FGr@MoS_2_ hybrids. The addition of 1.5 wt.% FGr@MoS_2_ resulted in a 60.9% reduction in the wear rate compared to neat CFRP. For the 1.2 wt.% FGr@MoS_2_-reinforced CFRP, the friction coefficient maintained a relatively steady value of approximately 0.46 at various temperatures, indicating frictional stability. However, the wear rate increased by 13.95% at 60 °C compared to that at room temperature. The interfacial bonding force between the FGr@MoS_2_ hybrid and the matrix, as well as the adhesive force with the surface of the counterpart ball, is improved, caused by the heterostructure of FGr@MoS_2_, resulting in enhanced mechanical properties and formation efficiency as well as the transfer film on the surface of the counterpart ball. The results suggest that an FGr@MoS_2_ micro-nano structure is a promising additive to be applied in polymer tribology.

## 1. Introduction

Carbon-fiber-reinforced polymer (CFRP) has been widely applied in key components of aerospace materials, vessels, and automobiles due to the high specific strength, corrosion resistance, and excellent designability of its structure and its performance [1,2,3,4,5]. The surface properties and the stress and strain distribution of CFRP can be modified by component design. As a result, polymer matrix composites can be used as sliding parts, such as bearings, seals, gears, etc. [6,7,8]. However, poor lubricity and low mechanical properties limit the further application of polymer matrix composites in the field of tribology.

Adding solid additives with various physical–chemical properties can effectively improve the tribological properties and temperature conductivity of polymer matrix composites [9,10,11]. The use of hard particles such as CNTs and SiO_2_ can achieve significant improvement in wear resistance by improving the hardness, elastic modulus, and strength of composites [12,13,14]. In addition, lubrication modification can be achieved by introducing two-dimensional materials such as graphite and MoS_2_ into the matrix due to their low shear strength.

Further research has shown that the use of single species of additives limited the improvement of composites in terms of surface characteristics and mechanical properties. Therefore, the synergistic effect of hybrids or different kinds of particles has attracted wide interest [15,16,17]. The addition of micro-BN and nano-Al_2_O_3_ achieved the effective improvement of the thermal conductivity of epoxy composites due to multi-scale and multi-performance characteristics [18]. The incorporation of alumina and nanoscale carbon fillers into polyimide can generally reduce the wear rate of composites by orders of magnitude [19]. The improved tribological characteristics of composites containing a polytype of particles are mainly attributed to the synergistic effect between various types of particles [20,21,22]. However, when the particles do not touch each other within the composite, the synergistic effect is usually not obvious because of the distance between each particle, especially at a low concentration of filler, which reduces the enhancement effect of the particles. Multi-particle hybridization can effectively address these deficiencies. Xu et al. [23] prepared a Fe_3_O_4_@MoS_2_ hybrid and found that the hybrid had the lubrication and anti-wear effects of the two types of particles. A CNF/MoS_2_ hybrid can significantly improve the lubricity and wear resistance of polymer-based composites. The analysis showed that the high strength of CNF and low interlayer shear characteristics of MoS_2_ cooperatively improved the self-lubrication and load-bearing performance of composites [24,25]. Reza Moghimi Monfared et al. [26] found that the use of MWCNT and nano-silica can improve the strength and abrasion resistance of fabric-reinforced epoxy composites.

In the above studies, the hybrid is usually the combination of hard particles and low interlaminar shear strength particles; as a result, the hybrid has the characteristics of both high-load-bearing and low-friction coefficients. Further studies have shown that the modification mechanism of hybrid-reinforced composites is complex and usually has multiple action modes [21,27]. However, current research has paid little attention to the hybrid that is synthesized by two different kinds of two-dimensional materials, and the action mechanism of hybrids is not clear. In this study, in order to investigate the synergistic effect of two-dimensional hybrids on polymer matrix composites during a sliding process, FGr@MoS_2_ hybrids (fluorinated graphite and MoS_2_) are synthesized by the hydrothermal method and used as additives to CFRP; then, the tribological properties of FGr@MoS_2_-hybrid-reinforced CFRP under dry sliding conditions at different temperatures are studied. The self-lubrication properties of CFRP and the potential mechanism are discussed.

## 2. Materials and Experiment

### 2.1. Materials

Here, 3K bi-directional, plain woven carbon fiber fabric (CF) (areal weight of 280 g/m^2^, thickness of 0.22 mm, and fiber diameter of 7 mm) was supplied by the Xi’an Aerospace Composite Research Institute. The silane coupling agent (KH-550) was purchased from Aladdin Industries. The epoxy (EPOLAM 2040) and hardener (EPOLAM 2042) were obtained from the Sika-Axson Group. The main physical and chemical parameters of CF and epoxy are listed in Table 1. Fluorinated graphite (FGr) (99.99%), with an average size of 50 μm, was purchased from the Sinopharm Group. Ammonium molybdate ((NH_4_)6Mo_7_O_24_∙4H_2_O) and thiocarbamide (CSN_2_H_4_) were purchased from the Tianjin Chemical Reagent Factory No.4 China Kaida Chemical Plant and Tianjin Tianli Chemical Reagent Co. LTD (Tianjin, China), respectively. All chemicals were used as received without further purification.

### 2.2. Surface Treatment of CF

In order to enhance the interfacial bonding strength between carbon fiber and matrix, the CF was treated using concentrated nitric acid: the CF was cut into 40 × 40 mm^2^ pieces, then immersed in concentrated nitric acid (65%) at 80 °C for 4 h, accompanied reflux condensation, followed by washing with distilled water until the pH of the cleaning solution was neutral. Finally, it was dried at 75 °C for 24 h [28].

### 2.3. Synthesis of FGr@MoS_2_ Hybrids

The surface treatment of FGr was performed to increase the dispersity of FGr within the solution and the binding force with MoS_2_. The specific process flow is as follows: firstly, 2 mg KH550 was added to a 50 mL mixture of distilled water and ethanol (1:9 by volume) with ultrasonic treatment; then, we adjusted the pH of the mixture to below 4.5 by adding oxalic acid; 40 mg FGr was added to the mixture after stirring for 1 h, followed by stirring for 4 h at 80 °C. Finally, the product was collected via vacuum filtration and then washed with ethyl alcohol and distilled water five times. The final precipitate was dried at 70 °C under vacuum [20].

Then, 120 mg (NH_4_)6Mo_7_O_24_∙4H_2_O and 210 mg CSN_2_H_4_ were added to 30 mL distilled water and stirred for 15 min at room temperature; after that, 30 mg FGr was added to the solution, followed by mechanical stirring. Then, suspensions were transferred into a 50 mL Teflon-lined stainless steel autoclave, sealed, and kept at 200 °C for 24 h. Then, the autoclave was cooled to room temperature naturally, and the product was collected through vacuum filtration, followed by washing with ethanol several times to remove any residue. Finally, the FGr@MoS_2_ hybrid was dried for 24 h at 70 °C under vacuum [21,22].

### 2.4. Preparation of CFRP

CFRP was fabricated by a vacuum-assisted resin transfer molding (VARTM) process [29]. A proper mass of FGr@MoS_2_ hybrids was added to 10 mL absolute ethyl alcohol and an ultrasonic instrument for 40 min. Next, the mixture was poured into 15 g epoxy, using a mechanical stirrer for 0.5 h, followed by ultrasonic for 0.5 h. After that, the mixture was placed in a vacuum oven to remove the solvent. After that, a 5 g hardener was added to the mixture using stirring. In this study, 5 plies of carbon fabric were used for the preparation of CFRP. The CFRP was cured for 24 h at 30 °C, followed by 15 h at 75 °C. For convenience, according to the proportion of FGr@MoS_2_ (wt.%) in the sample, the fabricated CFRP were designated as FGr@MoS_2_-0, FGr@MoS_2_-0.3, FGr@MoS_2_-0.6, FGr@MoS_2_-0.9, FGr@MoS_2_-1.2, and FGr@MoS_2_-1.5, respectively.

### 2.5. Test and Characterization Technique

**Figure 1:**
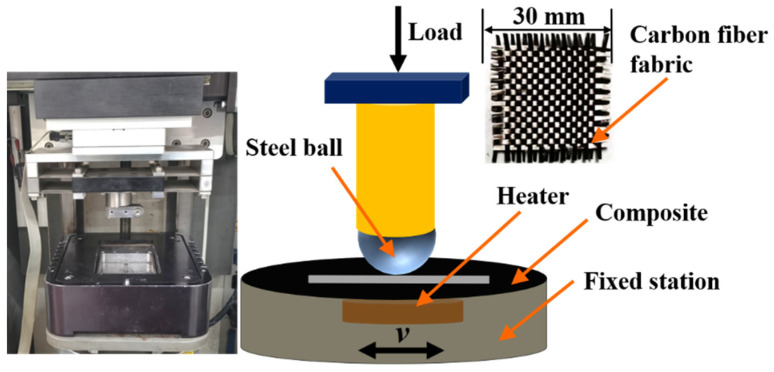
Test principle of dry sliding.

The tribological performances of CFRP were evaluated by using a UMT-2 ball-on-disk tribometer (CETR-2, Campbell, CA, USA). The reciprocating motion, with a sliding length of 6 mm as the sliding mode, and the testing schematic diagram are shown in Figure 1. A heating device was placed under the platform to achieve different temperatures. A GCr15 steel bearing ball, with a diameter of 9.6 mm and a surface roughness of Ra = 0.22, was used as the sliding counterpart and the size of the sample was 30 × 30 mm^2^. The friction tests were performed at a sliding frequency of 2 Hz for 30 min, with an applied load of 2 N under different temperatures (23 ± 1, 40 ± 1, and 60 ± 1 °C); the ambient humidity was approximately 30 ± 2%. The ball and sample were cleaned with acetone prior to testing. Each test was repeated three times, and the average value was used as the final result.

The crystal phase structure of FGr@MoS_2_ hybrids was obtained by X-ray diffraction measurements (D8 Advance A25 at 40 kV and 40 mA with Cu Ka). The detailed morphologies and microstructures of FGr@MoS_2_ hybrids were conducted using scanning electron microscopy (SEM, Gemini SEM 500 at 10 kV). The component structure of the FGr@MoS_2_ hybrids was obtained through a Raman spectrometer (Raman, Thermo Fisher, Waltham, MA, USA, DXR2xi, 512 nm Ar laser). The wear volume and worn morphologies of the sample were measured and evaluated using a laser scanning confocal microscope (Olympus, Japan, OLS4000) and a digital microscope (Shanghai Wanheng Precision Instrument, Shanghai, China, MM-158V).

## 3. Results and Discussion

### 3.1. Microstructure of FGr@MoS_2_ Hybrids

**Figure 2:**
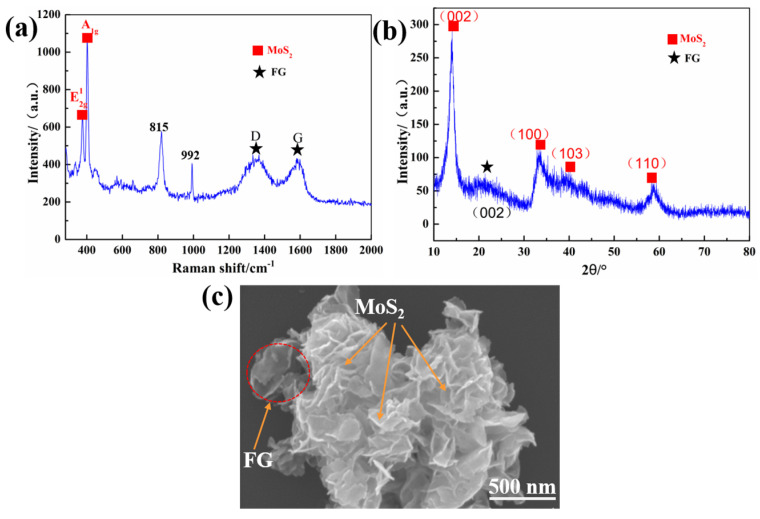
(**a**) Raman spectrum of FGr@MoS_2_ hybrid, (**b**) XRD pattern of FGr@MoS_2_ hybrid, and (**c**) SEM image of FGr@MoS_2_ hybrid.

Figure 2 shows the microstructural characteristics, crystal structure, and chemical composition of FGr@MoS_2_ hybrids. It can be seen from Figure 2a that two characteristic peaks, A_1g_ and E_2g_ on the Raman spectra, are located near 383.2 and 403.7 cm^−1^, respectively, which belongs to the characteristic peaks of MoS_2_, indicating the generation of MoS_2_ and that the hydrothermal reaction does not destroy the structure of the FGr [30]. The peaks located at 815 and 992 cm^−1^ in the Raman spectra indicate the generation of a very small amount of MoO_3_ in the as-grown MoS_2_ [31]. The XRD pattern is shown in Figure 2b; the main diffraction peak of 002 is located at 14.6°, which also indicates the formation of MoS_2_ in the hybrid [32]. It can be observed that the nano-sheet MoS_2_ is closely and evenly attached to the surface of the FGr to form the hybrid in a micro-nano structure.

### 3.2. Effect of Content of FGr@MoS_2_ on the Tribological Properties of CFRP

**Figure 3:**
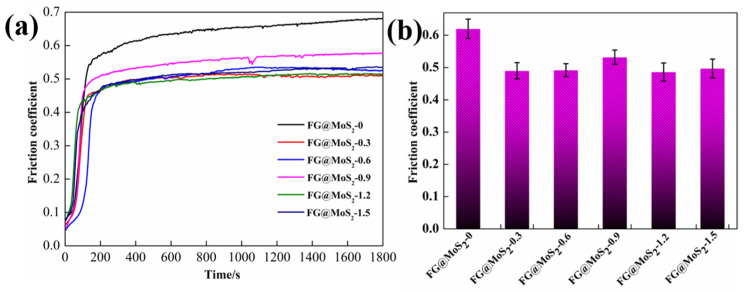
(**a**) Real-time friction curve and (**b**) average friction coefficient of CFRP containing different contents of FGr@MoS_2_ hybrid.

The friction coefficients of CFRP with different FGr@MoS_2_ hybrid contents are shown in Figure 3. From Figure 3a, it can be seen that the real-time friction coefficients of six types of samples increase rapidly at first and then tend to be stable. In the initial sliding stage, the surface of the composite is in its original condition, with low surface roughness; then, the surface of the composite starts to be destroyed in a short time under the high contact pressure and sliding shear force, which leads to increased friction coefficients. As the sliding continues, the steel ball contacts the carbon fiber further, the transfer film on the surface of the steel ball is generated, and the destruction of the composite tends to be stable under the synergism of the matrix and carbon fiber. The composite thus reaches a relatively stable friction coefficient. Furthermore, it can be found that the ranges of the friction coefficient decrease with the addition of FGr@MoS_2_ hybrids, showing enhanced stability. From Figure 3b, the presence of FGr@MoS_2_ improves the lubricating property of the composite. The friction coefficient increases with increased FGr@MoS_2_ content; the friction coefficient of CFRP reached the maximum value of about 0.56 when FGr@MoS_2_ content was 0.9 wt.%. As the content of FGr@MoS_2_ increased to 1.2 wt.%, the friction coefficient achieved the minimum value of 0.48. However, when the FGr@MoS_2_ content further increased to 1.5 wt.%, the lubrication performance of the composite deteriorated.

**Figure 4:**
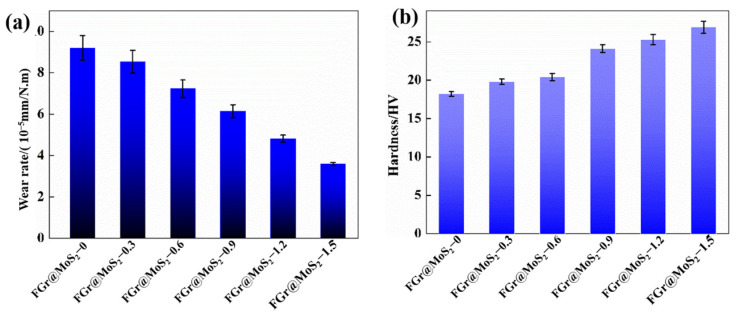
(**a**) Wear rate and (**b**) Vickers hardness of CFRP containing different contents of the FGr@MoS_2_ hybrid.

Figure 4 shows the wear rates and Vickers hardness of six kinds of CFRP. It is obvious that the wear rate of CFRPs without FGr@MoS_2_ hybrids reaches the maximum of 9 × 10^−4^ mm^3^/N·m. With the addition of FGr@MoS_2_, the wear resistance of the CFRP is significantly improved, and with the increase in FGr@MoS_2_ content, the wear resistance of the composite is continuously enhanced. When the FGr@MoS_2_ content reached 1.5 wt.%, the wear rate of CFRP reached the lowest value of 3.6 × 10^−4^ mm^3^/(N·m), which was 60.9% lower than that of the CFRP without the FGr@MoS_2_ hybrid. In addition, it can be seen that the harness of the CFRP achieved enhancement with the addition of FGr@MoS_2_ and reached the maximum value when the content of hybrids was increased to 1.5 wt.%.

**Figure 5:**
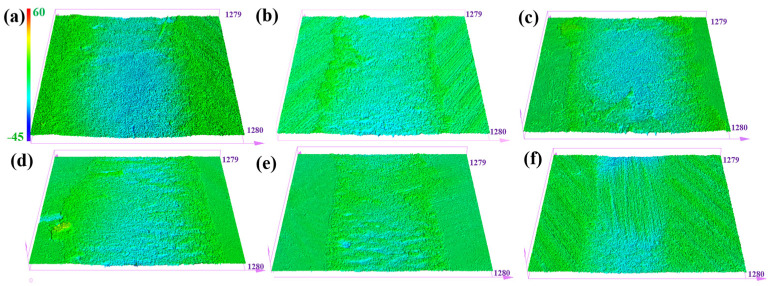
3D morphologies of the worn surface of: (**a**) FGr@MoS_2_-0; (**b**) FGr@MoS_2_-0.3; (**c**) FGr@MoS_2_-0.6; (**d**) FGr@MoS_2_-0.9; (**e**) FGr@MoS_2_-1.2; (**f**) FGr@MoS_2_-1.5.

Figure 5 shows the 3D morphology of the worn surface for different types of CFRPs. The obvious border of the wear track can be found when the FGr@MoS_2_ content is less than 0.9 wt.% and the surface roughness is relatively high, which corresponds to a higher wear volume. Furthermore, the use of 1.2 wt.% FGr@MoS_2_ can achieve a significant reduction in wear depth and surface roughness of the CFRP, and the addition of 1.5 wt.% FGr@MoS_2_ causes a further decrease in wear depth. It can be concluded that the tribological properties of FGr@MoS_2_-1.5 are better than those of other groups of samples.

**Figure 6:**
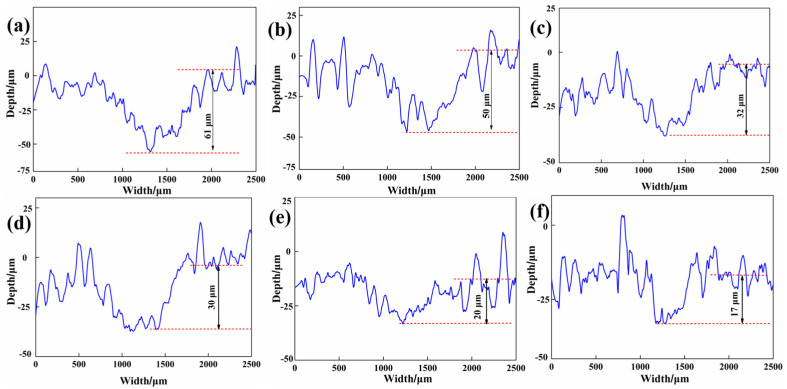
Wear track profiles of CFRP containing different contents of FGr@MoS_2_: (**a**) FGr@MoS_2_-0; (**b**) FGr@MoS_2_-0.3; (**c**) FGr@MoS_2_-0.6; (**d**) FGr@MoS_2_-0.9; (**e**) FGr@MoS_2_-1.2; (**f**) FGr@MoS_2_-1.5.

Figure 6 shows the wear track depth of six kinds of CFRP. It can be found that the wear track depth decreases with increasing FGr@MoS_2_ content. When 1.5 wt.% FGr@MoS_2_ hybrids were added into CFRP, the CFRP showed the lowest wear track depth of 17 μm, which was reduced by approximately 72.1% compared to that of FGr@MoS_2_-0. Meanwhile, it can be found that the wear profiles of the six types of CFRP show a large fluctuation, which is mainly caused by the matrix and the fiber peeling off, which indicates that the mechanical properties of the matrix play a very important role in the dry friction performances of CFRP.

**Figure 7:**
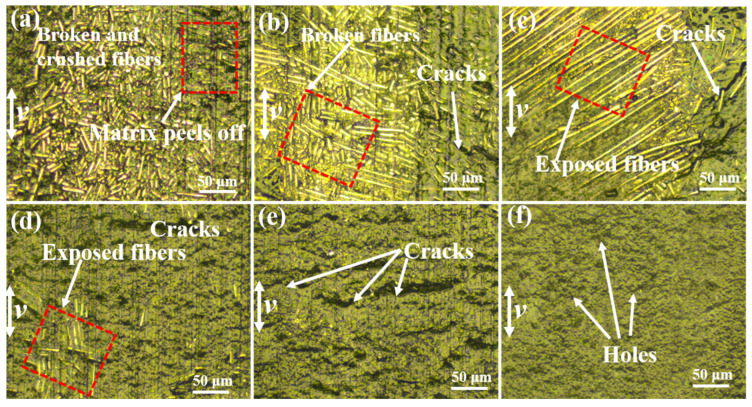
Optical images of worn surface of composites containing different contents of FGr@MoS_2_ under room temperature: (**a**) FGr@MoS_2_-0; (**b**) FGr@MoS_2_-0.3; (**c**) FGr@MoS_2_-0.6; (**d**) FGr@MoS_2_-0.9; (**e**) FGr@MoS_2_-1.2; (**f**) FGr@MoS_2_-1.5.

Optical images of the worn surface of CFRP with different contents of FGr@MoS_2_ are shown in Figure 7. From Figure 7a, it can be observed that matrix loss has occurred on the surface of hybrid FGr@MoS_2_-0, exposing a number of carbon fibers, which break into small fragments, indicating severe wear. The elastic and strain mismatch between the matrix and the carbon fiber is considered to be the main reason for CFRP wear. The polymer matrix is prone to plastic failure due to its low mechanical properties under a reciprocating shear force. As the matrix breaks and peels off, the steel ball comes into further contact with the carbon fiber, which has high strength, high brittleness, and poor lubrication properties, resulting in the appearance of a carbon fiber fracture and, hence, an increased friction coefficient. In this case, the wear mechanism is mainly dominated by furrow wear. As shown in Figure 7b, the addition of FGr@MoS_2_ leads to an increase in the size of the broken carbon fiber on the CFRP surface, indicating enhanced wear resistance compared to FGr@MoS_2_-0. From Figure 7c, it is clear that the exposed fibers are on the surface of composites; nevertheless, the amount of broken fibers is not found. As shown in Figure 7d,e, the width of the wear track decreases with increasing FGr@MoS_2_ content, and large areas of fibers are not observed; only the broken fibers are present on the CFRP surface. Furthermore, it can be seen from Figure 7f, when the FGr@MoS_2_ content reaches 1.5 wt.%, there is no obvious debris accumulation at the margin of the wear track, the large area of the matrix peels off does not occur, and local smooth areas can be found on the surface of the sliding zone, all of which indicate improved anti-wear and lubricity.

The low FGr@MoS2 level in the composite leads to a low formation efficiency of the transferred film on the surface of the counterpart ball. In this case, the mechanical properties of the epoxy matrix and CF have important influences on the tribological properties of CFRP. The matrix is more prone to peeling than the carbon fiber due to its low mechanical properties, and then the steel ball comes into contact with the exposed carbon fiber, resulting in a high friction coefficient due to the poor lubrication of the carbon fiber. The CFRP possesses enhanced hardness and strength after the addition of FGr@MoS_2_. As a result, the CFRP exhibits an increased wear resistance. Furthermore, the increased mechanical properties of the matrix reduce the performance difference between the matrix and the carbon fiber, leading to a more congruous distribution of stress and deformation within the CFRP, which is also responsible for the enhancement in wear resistance.

**Figure 8:**
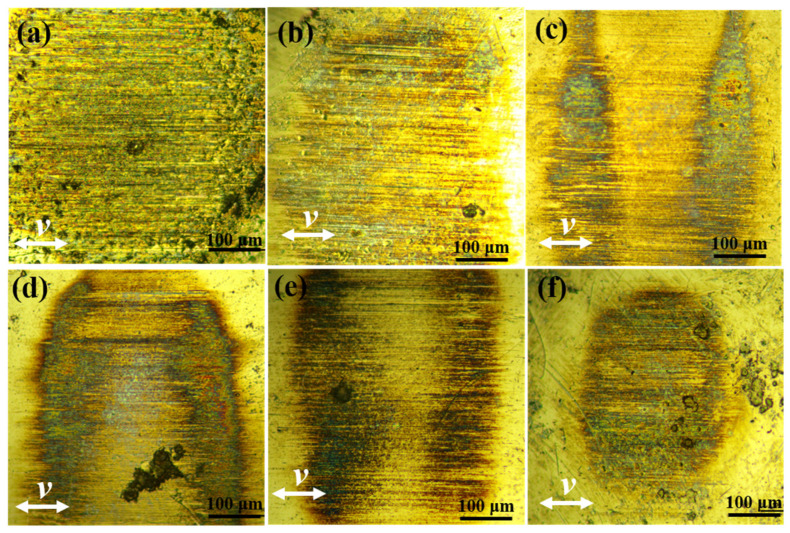
Worn surfaces of counterpart ball sliding against: (**a**) FGr@MoS_2_-0; (**b**) FGr@MoS_2_-0.3; (**c**) FGr@MoS_2_-0.6; (**d**) FGr@MoS_2_-0.9; (**e**) FGr@MoS_2_-1.2; (**f**) FGr@MoS_2_-1.

The wear morphologies of the counterpart balls are shown in Figure 8. From Figure 8a, less transfer film on the surface of the counterpart ball was observed with the CFRP without the FGr@MoS_2_ hybrid, and many deep scratches were found on the surface of the ball. The reason can be attributed to the direct contact between the counterpart ball and the carbon fiber, resulting in the severe wear of the counterpart and a high friction coefficient because of the poor lubricity of carbon fiber. From Figure 8b, it can be found that the width and depth of scratches decreased with the addition of the FGr@MoS_2_ hybrid; the main reason should be the improved transfer film after using fillers. From Figure 8c,f, the continuous and uniform transfer film on the surface of the counterpart ball can be observed, and the wear track width decreases with the increase in FGr@MoS_2_ hybrid content, indicating enhanced tribological properties. With the increased concentration of FGr@MoS_2_ hybrids, the formation efficiency and quality of the transfer film on the surface of the counterpart ball are improved due to the strong adhesive force of MoS_2_ and metal. Then, the wear mechanism changes from furring wear to adhesive wear, and the friction coefficient mainly depends on the interlaminar shear force and bearing capacity of the transfer film. Thus, the CFRP shows a decreased friction coefficient because of the low interlayer shear force of FGr and MoS_2_ [30,33,34].

### 3.3. Effect of Temperature on the Tribological Properties of CFRP

**Figure 9:**
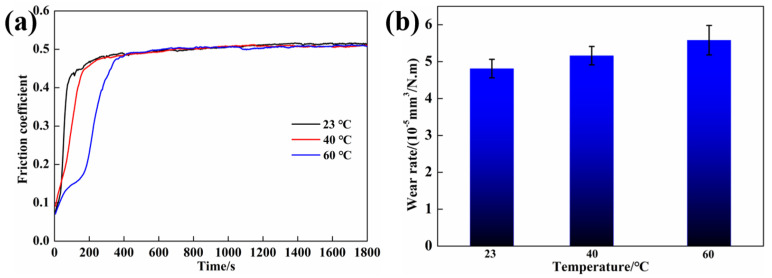
Tribological properties of FGr@MoS_2_-1.2 at different temperatures: (**a**) friction coefficient and (**b**) wear rate.

Due to the poor thermal conductivity of polymers, instantaneous heat is more likely to accumulate in polymer matrix composites under dry sliding conditions, which is a significant cause of severe wear in polymer matrix composites. Therefore, the effect of temperature on the tribological properties of FGr@MoS_2_-1.2 was further investigated, and the results are shown in Figure 9. It can be seen from Figure 9a that the running-in time of FGr@MoS_2_-1.2 increases with an increase in temperature, and the CFRP presents an almost identical friction coefficient of about 0.46 at different temperatures. The main reason can be ascribed to the sharp decline in the hardness and strength of the matrix, caused by the rising temperature of the contact zone, which reduces the contact time between the matrix and the counterpart ball, reducing the formation efficiency of the transfer film on the surface of the steel ball and, therefore, increasing the running-in time. For the friction coefficient, the contact area between the steel ball and the carbon fiber increases due to the destruction of the matrix; the mechanical properties of the carbon fiber are less affected by temperature, so FGr@MoS_2_-1.2 shows a similar friction coefficient at different temperatures. However, it can be seen from Figure 9b that the wear rate increases with an increase in temperature. The temperature gradually reaches the glass transition temperature of epoxy resin and softens the matrix as the heat accumulates, resulting in reduced wear resistance.

**Figure 10:**
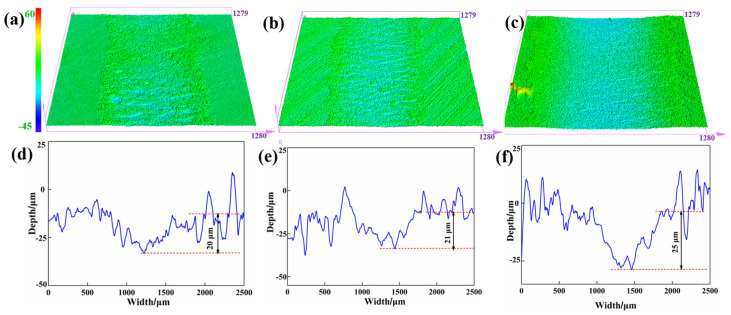
Three–dimensional morphologies of the worn surface of FGr@MoS_2_-1.2 at (**a**) 23 °C, (**b**) 40 °C, and (**c**) 60 °C. Wear track profiles of MoS_2_-1.2 at (**d**) 23 °C, (**e**) 40 °C, and (**f**) 60 °C.

Figure 10 shows the wear morphologies and profiles of FGr@MoS_2_-1.2 at different temperatures. From Figure 10a–c, the CFRP shows increasingly apparent wear tracks with increasing temperature, indicating the decreased wear resistance. The CFRP shows a slight increase in wear depth as the temperature was increased to 40 °C (See Figure 10d,e). Additionally, from Figure 10d,f, the wear depth increased by 25% when the temperature was raised to 60 °C compared with that at room temperature. The reduction in the mechanical properties and bearing capacity of the polymer matrix at the high temperature is considered to be the main cause of the reduced wear resistance.

**Figure 11:**
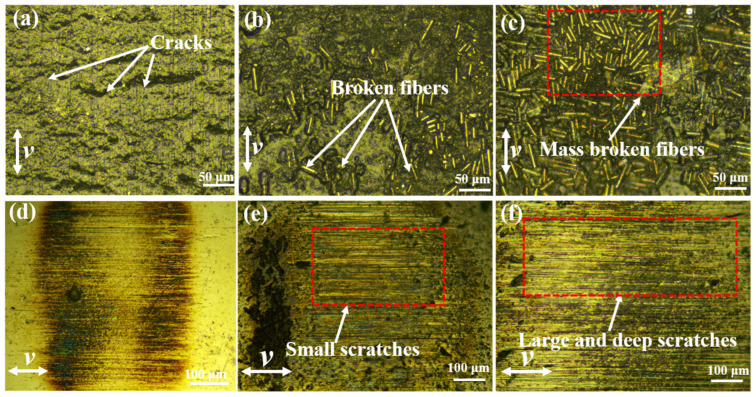
Optical images of worn surface of FGr@MoS_2_-1.2 at (**a**) 23 °C, (**b**) 40 °C, and (**c**) 60 °C; counterpart ball at (**d**) 23 °C, (**e**) 40 °C, and (**f**) 60 °C.

Figure 11 shows the microscopy of the worn surfaces of FGr@MoS_2_-1.2 and the counterpart ball at different temperatures. It can be found that the width of the wear track increases when the temperature is increased to 40 °C, indicating decreased wear resistance. When the temperature was further increased to 60 °C, the wear track width increased significantly, and a large amount of bare fibers could be observed, showing relatively severe damage.

### 3.4. Tribological Mechanism

**Figure 12:**
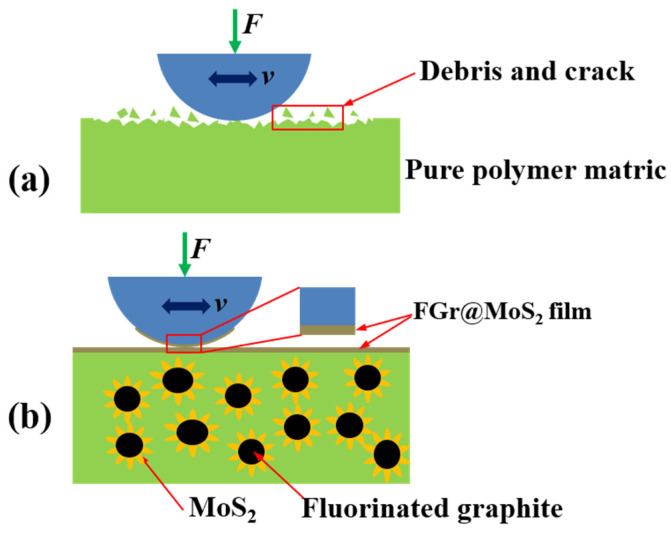
Friction mechanism of CFRP: (**a**) without hybrid; (**b**) containing hybrid.

Figure 12 shows the wear mechanism of FGr@MoS_2_-hybrid-reinforced CFRP. Pure epoxy matric CFRP is easily to break and fall off because of the low bearing capacity and poor wear resistance under the shear stresses (Figure 12a). When FGr and MoS_2_ are used as hybrids in CFRP, the roughness of the additive increases as the deposition of flaky MoS_2_ on the surface of the FGr increases, leading to an increase in the bonding force between the hybrid and the matrix. Therefore, the external load can be effectively transferred from the matrix to the carbon fiber, with the result that CFRP shows improved load-bearing properties and wear resistance. On the other hand, FGr@MoS_2_ hybrids are more likely to adhere to the surface of steel balls due to the strong binding force of MoS_2_ and the metal counterpart [35,36]. Moreover, Figure 12b also shows that the MoS_2_ generated by a hydrothermal reaction can be uniformly dispersed on the surface of the FGr, which improves the dispersion efficiency and stability of MoS_2_ in the matrix indirectly and, thus, improves the formation efficiency and quality of the transfer film. As a result, the CFRP exhibits improved self-lubrication properties and wear resistance.

## 4. Conclusions

In this study, the FGr@MoS_2_ hybrids were synthesized and used as lubricating additives to improve the tribological properties of CFRP under dry sliding conditions at different temperatures. The effect of hybrid contents and ambient temperature on the tribological properties of CFRP were investigated, and related mechanisms were illustrated. The main conclusions are drawn as follows:

1. FGr@MoS_2_ hybrids with micro-nano architecture were successfully prepared by the hydrothermal method, and the hybrids possess the high specific surface area and good dispersion of MoS_2_.
2. CFRP showed improved tribological performances when FGr@MoS_2_ was used as a lubricant additive. The CFRP showed the lowest friction coefficient of 0.48 when the hybrid content was 1.2 wt.%, and the CFRP achieved the lowest wear rate of 3.59 × 10^−4^ mm^3^/(N·m) when the hybrid content was 1.5 wt.%. The deposition of MoS_2_ on the surface of the FGr improves the binding force between the additives and the matrix as well as the efficient formation of the transfer film, thus contributing to an enhanced decrease in wear and lubrication.
3. The 1.2 wt.% FGr@MoS_2_-reinforced CFRP showed good friction stability due to the high temperature resistance of FGr. Additionally, the wear rate increases slightly with the increase in temperature, indicating a potential engineering application prospect.

## Figures and Tables

**Table 1:** Characteristic parameters of CF and epoxy.

Materials	Elasticity Modulus (GPa)	Tensile Strength (MPa)	Breaking Elongation (%)
CF	225	3500	1.5
Epoxy	3.2	75	8.7

## Data Availability

The data presented in this study are available on request from the corresponding author.
